# Evaluating the quality and reliability of youtube videos on Hirschsprung's disease: a comprehensive analysis for patients, parents and health professionals

**DOI:** 10.1007/s00383-025-06064-0

**Published:** 2025-06-17

**Authors:** Gül Doğan, Hülya İpek

**Affiliations:** 1 Department of Pediatric Surgery, Hitit University ErolOlcok Training and Research Hospital, Çepni Mahallesi, İnönü Cd. No:176, Çorum, 19200 Turkey; 2 Department of Pediatric Surgery, Hitit University Faculty of Medicine, Çorum, Turkey

**Keywords:** Hirschsprung's disease, Congenital megacolon, YouTube™, Video analysis

## Abstract

**Purpose:**

This study aims to evaluate the quality and reliability of the informational and surgery-oriented content of YouTube videos on Hirschsprung's disease.

**Methods:**

On October 1, 2024, a YouTube search was conducted on Hirschsprung’s disease, and 60 videos with over 10,000 views were included in the study. The reliability and quality of the videos were assessed by two independent pediatric surgeons using the Global Quality Scale (GQS), Modified DISCERN (mDISCERN), and American Medical Association (JAMA) scales. VPI was calculated as (like ratio × view ratio)/100, where view ratio refers to the number of views per day since the video's upload date.

**Results:**

Of the 60 videos, 53 (88.3%) were informational, while 9 (11.7%) were surgery-oriented. Videos were uploaded by private hospitals (15%), physicians (31.7%), and private youtube channels (53.3%). Thirteen videos (21.7%) utilized animations. The mean video length was 640.5 ± 729.8 s, with an average view count of 83,728 ± 265,503 and a mean VPI of 49.8 ± 157.4. The average mDISCERN score was 2.53 ± 0.98, the GQS score was 2.52 ± 1.2, and the JAMA score was 2.55 ± 0.53. Although weak, statistically significant positive correlations were observed between VPI and quality scores (mDISCERN: *r* = 0.32, *P* = 0.014; GQS: *r* = 0.26, *P* = 0.048; JAMA: *r* = 0.34, *P* = 0.008), suggesting that more popular videos tended to be of slightly better quality. Older videos were associated with lower quality scores (*P* < 0.05).

**Conclusion:**

This study revealed that YouTube videos related to Hirschsprung's disease are generally of low quality. This situation increases the risk of patients and their families being exposed to misleading or incomplete information.

## Introduction

Hirschsprung’s disease (HSCR) is a congenital disorder characterized by the absence of nerve cells (ganglion cells) in parts of the intestines, preventing normal bowel movements [1, 2]. This condition typically presents in newborns with bowel obstruction symptoms [3]. Diagnosis is made through clinical symptoms, radiological imaging, and biopsy results [3, 4]. The primary treatment is surgical, involving the removal of the affected section of the bowel [1]. Hirschsprung’s disease affects approximately 1 in 5000 live births, and without timely intervention, it can lead to severe health complications [5].

With the widespread use of the internet and social media, both patients and healthcare professionals have gained unprecedented access to health-related information [6]. YouTube has become a significant platform for sharing medical content, including surgical videos, which offer valuable insights to both patients, caregivers, and medical professionals [7]. However, the growing reliance on online resources brings challenges regarding the accuracy and reliability of these contents [8]. The surge in health-related information available on the internet has emphasized the need to scrutinize the quality of these sources, ensuring that users access accurate and trustworthy information [9]. Recent studies evaluating pediatric health topics including strabismus surgery [10], infantile colic [11], adenotonsillectomy [12], and stoma care [13] further demonstrate that YouTube videos often lack accuracy, completeness, or adherence to medical guidelines, reinforcing the need for critical evaluation of online health content.

With the growing reliance on YouTube for medical information, particularly for complex conditions like Hirschsprung's disease, it becomes essential to question the accuracy and quality of the content shared. For a disease that requires precise diagnosis and treatment, it is critical to determine whether the information provided in these videos is accurate and reliable [9]. Are the most viewed videos truly high-quality, or are they merely popular due to accessibility or presentation? Additionally, should patients, caregivers, and even healthcare professionals focus on highly viewed videos, or should they prioritize content shared by medical experts and institutions? This raises concerns about whether view count correlates with content quality and whether it’s safe for users to rely on popular videos for critical health information [8, 9, 14]. Thus, evaluating the quality of these videos becomes a necessary step in ensuring that individuals accessing this information, especially those affected by Hirschsprung's disease, are guided towards accurate, evidence-based knowledge. The purpose of this study is to analyze the content, reliability, and quality of YouTube videos related to Hirschsprung’s disease. By evaluating the effectiveness of these videos in delivering accurate information, we aim to contribute to establishing a standard for assessing the quality of medical content on online platforms, benefiting both patients and healthcare professionals.

## Materials and methods

### Data collection

In this study, videos obtained from YouTube as of October 1, 2024, were statistically analyzed. A search was conducted on YouTube using keywords such as "Hirschsprung’s disease" and "congenital megacolon," either individually or in various combinations. Different spelling strategies related to the disease (e.g., Hirschsprung or Hirschprung, Hirschsprung’s or Hirschprung’s) were also considered during the search. The number of videos with irrelevant content was 2, the number of videos shorter than 20 s was 1, and the number of sponsored or promoted videos was 3. In total, 6 videos were excluded from the analysis. The search results were ranked based on view counts, and 60 videos with more than 10,000 views related to Hirschsprung's disease were included in the study. To ensure inclusion of videos with meaningful viewer exposure, a threshold of 10,000 views was applied. This criterion was informed by a recent study by Coşkun and Demir (2024), which applied a similar cutoff in a YouTube content quality analysis on circumcision [15]. Similarly, Aktar Uğurlu and Uğurlu (2025) used the same threshold in their study analyzing YouTube videos about tympanostomy tubes [16]. Furthermore, this threshold aligns with YouTube's own benchmark for audience engagement and monetization eligibility, supporting its validity as a marker of content visibility. Focusing on highly viewed videos allowed us to evaluate content with greater public health relevance and viewer influence potential.

### Evaluation criteria

In this cross-sectional study, the reliability and quality of YouTube videos were evaluated using the Global Quality Scale (GQS), Modified DISCERN, and American Medical Association (JAMA) scales. Two independent pediatric surgery specialists assessed all videos using the GQS, Modified DISCERN, and JAMA scales. Additionally, the view rate, like ratio, and Video Power Index (VPI) values of the videos were calculated to determine their popularity.

The Global Quality Scale (GQS) was developed by Bernard et al. (2007) to evaluate the usefulness of medical videos for patients. This scale assesses the scientific accuracy of the videos, effective communication skills, and the benefits offered to the audience. It is scored between 1 and 5, where a score of 1 indicates low quality and lack of usefulness, and a score of 5 indicates high quality and usefulness [17].

The Modified DISCERN Scale is an abbreviated and adapted version of the DISCERN scale, originally developed by Charnock et al. (1999) to assess the reliability of health-related information sources [18]. Singh et al. (2012) restructured it into a 5-item format. The scale scores each criterion as 1 (yes) or 0 (no), giving a total score ranging from 0 to 5; a higher score indicates a higher quality video [19].

The JAMA Video Quality Analysis, developed by Silberg et al. (1997), evaluates the content of medical videos. It consists of four criteria: authorship, which assesses whether the video clearly identifies the author and if the individual is qualified to share medical information; attribution, which examines whether the information provided is based on reliable sources and proper attributions are made; disclosure, which evaluates if the video reveals potential conflicts of interest or sponsorship information; and currency, which considers whether the information presented in the video is up to date. These criteria assess both the scientific accuracy and the capacity of the video to provide reliable information to the viewer. Each criterion is scored on a scale of 1, with a total score ranging from 0 to 4. Higher scores indicate a more reliable video [20].

The Video Power Index (VPI) is a metric developed by Erdem et al. (2018) that measures the popularity and viewer engagement of videos on social media platforms. VPI calculations are based on the video’s view count, like count, dislike count, and the time elapsed since the video was uploaded. The formulas are as follows: View ratio = ([view count] / [days since upload]), Like ratio = ([100 × like count] / [like count + dislike count]), and Video Power Index = (Like ratio × view ratio / 100) [21].

### Statistical analysis

All statistical analyses were performed using SPSS software (Version 22.0, SPSS Inc., Chicago, IL, USA, licensed by Hitit University). Categorical variables were described as frequencies (*n*) and percentages (%). Continuous variables were reported as mean ± standard deviation for normally distributed data and as median and range (min–max) for non-normally distributed data. The distribution of the data was evaluated using the Kolmogorov–Smirnov or Shapiro–Wilk tests, depending on the sample size, as well as graphical methods such as Q-Q plots and histograms; these evaluations were taken into account in the selection of appropriate statistical tests. Since the assumptions of parametric tests were not met, comparisons between two independent groups were performed using the Mann–Whitney *U* test, and comparisons between more than two independent groups were made using the Kruskal–Wallis test. When the Kruskal–Wallis test indicated significant differences, pairwise comparisons were conducted using Dunn-Bonferroni post-hoc tests. Correlations between numerical variables were evaluated using the Spearman correlation coefficient. Inter-rater agreement between the two independent raters was examined using the intraclass correlation coefficient (ICC), interpreted as follows: below 0.5 indicates poor agreement, 0.5–0.75 indicates moderate agreement, 0.75–0.9 indicates good agreement, and above 0.9 indicates excellent agreement. A statistical significance level of *P* < 0.05 was considered significant.

## Results

Of the 60 videos on Hirschsprung’s disease, 53 (88.3%) were intended to provide information, and 7 (11.7%) were surgery-related. The videos were uploaded by private hospitals (*n* = 9, 15%), physicians (*n* = 19, 31.7%), and private YouTube channels (*n* = 32, 53.3%). Thirteen videos (21.7%) utilized animations. The average values for video length, time since the videos were uploaded, view count, and Video Power Index (VPI) were 640.5 ± 729.8 s, 2,355 ± 1,550 days, 83,728 ± 265,503, and 49.8 ± 157.4, respectively. Other statistical findings related to the videos are presented in Table 1.

**Table 1:** Descriptive statistical results of the characteristics of the YouTube videos analysed

	**n**	**%**
**Video content**		
Informational Video	53	88.3
Surgical video	7	11.7
**Video sources**		
Private Hospital	9	15
Physician	19	31.7
Private YouTube Channel	32	53.3
**Expression with animation**		
No	47	78.3
Yes	13	21.7
	**Mean ± SD**	**Median (min–max)**
**Video features**		
Video length (seconds)	640.5 ± 729.8	404 (41–4,339)
Time since upload (days)	2,355 ± 1,550	1,841 (299–6,345)
Number of views	83,728 ± 265,503	25,383 (10,273–2043,258)
Number of likes	661.4 ± 966	387 (11–4,445)
Number of dislikes	16.2 ± 38.6	4.5 (0–257)
Comments	41.92 ± 56.4	21.5 (2–283)
View ratio	51.8 ± 167.73	18 (2–1,289)
Like ratio	96.93 ± 5.42	99 (64–100)
VPI	49.76 ± 157.43	17.7 (1.7–1,207)
**Scales**		
Modified DISCERN	2.53 ± 0.98	3 (1–4)
GQS	2.52 ± 1.2	2 (1–5)
JAMA	2.55 ± 0.53	3 (1–3)

*VPI* Video Power Index, *GQS* global quality scale, *JAMA* Journal of the American Medical Association

The inter-rater reliability, measured by intraclass correlation coefficients (ICC), indicated excellent agreement between two independent pediatric surgery specialists for the mDISCERN, GQS, and JAMA scores (ICC: 0.918, ICC: 0.912, ICC: 0.928, respectively, *P* < 0.001). The mean scores for mDISCERN, GQS, and JAMA were 2.53 ± 0.98, 2.52 ± 1.2, and 2.55 ± 0.53, respectively. According to the GQS, 51.7% (*n* = 31) of the 60 videos were rated as low quality, 21.7% (*n* = 13) as medium quality, and 26.7% (*n* = 16) as high quality. Only 3 videos (5%) received a full score of 5 on the GQS scale, and no videos received full scores on the mDISCERN or JAMA scales.

The most viewed and highest-ranked video by VPI (2,043,258 views, VPI score of 1,207) was about anorectal manometry in the diagnosis of Hirschsprung’s disease. Uploaded in 2020, the video had mDISCERN, GQS, and JAMA scores of 2, 1, and 2, respectively. The second most viewed and popular video, uploaded in 2021 and covering rectal irrigations (299,070 views, VPI: 242), had scores of 2, 1, and 2, respectively. The third most viewed video, uploaded in 2019, addressed colitis and fecal incontinence (259,855 views, VPI: 134), with scores of 4, 4, and 3, respectively.

**Fig. 1:**
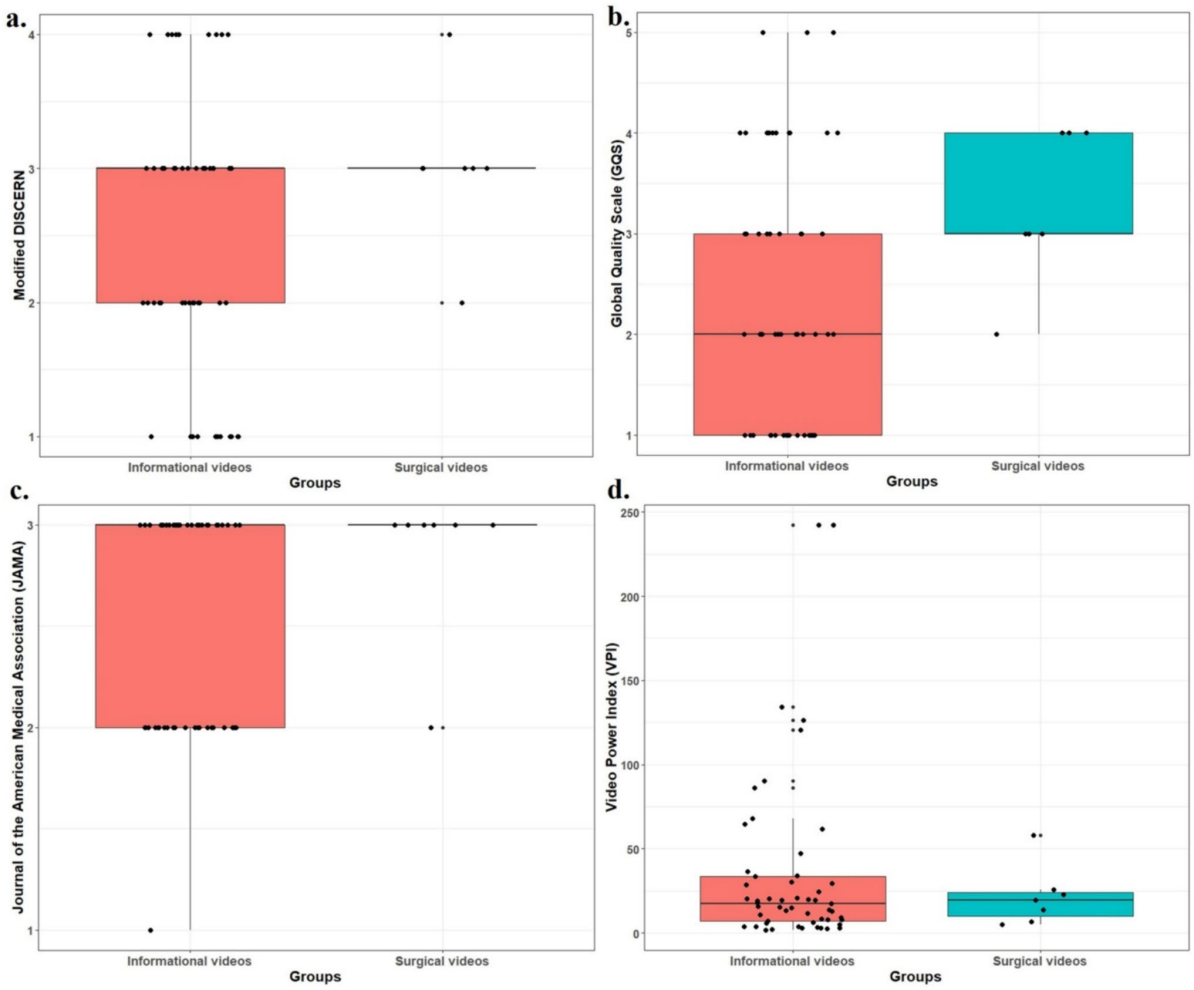
Boxplot with jitter showing the distribution of Global Quality Scale (GQS), Modified DISCERN, Journal of American Medical Association (JAMA) and Video Power Index (VPI) scores between informative and surgical videos

As normality was not met between informational and surgery-related video groups (mDISCERN: *P* < 0.001,* P* = 0.024; GQS: *P* < 0.001, *P* = 0.086; JAMA: *P* < 0.001, *P* < 0.001), group comparisons were made using the Mann–Whitney U test. Although surgery-related videos had higher mDISCERN, GQS, and JAMA scores (3 ± 0.58, 3.29 ± 0.76, 2.86 ± 0.38) than informational videos (2.47 ± 1.01, 2.42 ± 1.28, 2.51 ± 0.54), the differences were not significant (*P* = 0.202, *P* = 0.072, *P* = 0.164, respectively; Fig. 1). Likewise, no significant differences were found in viewing duration, views, likes, or VPI scores (*P* = 0.415, *P* = 0.119, *P* = 0.604, *P* = 0.946).

**Fig. 2:**
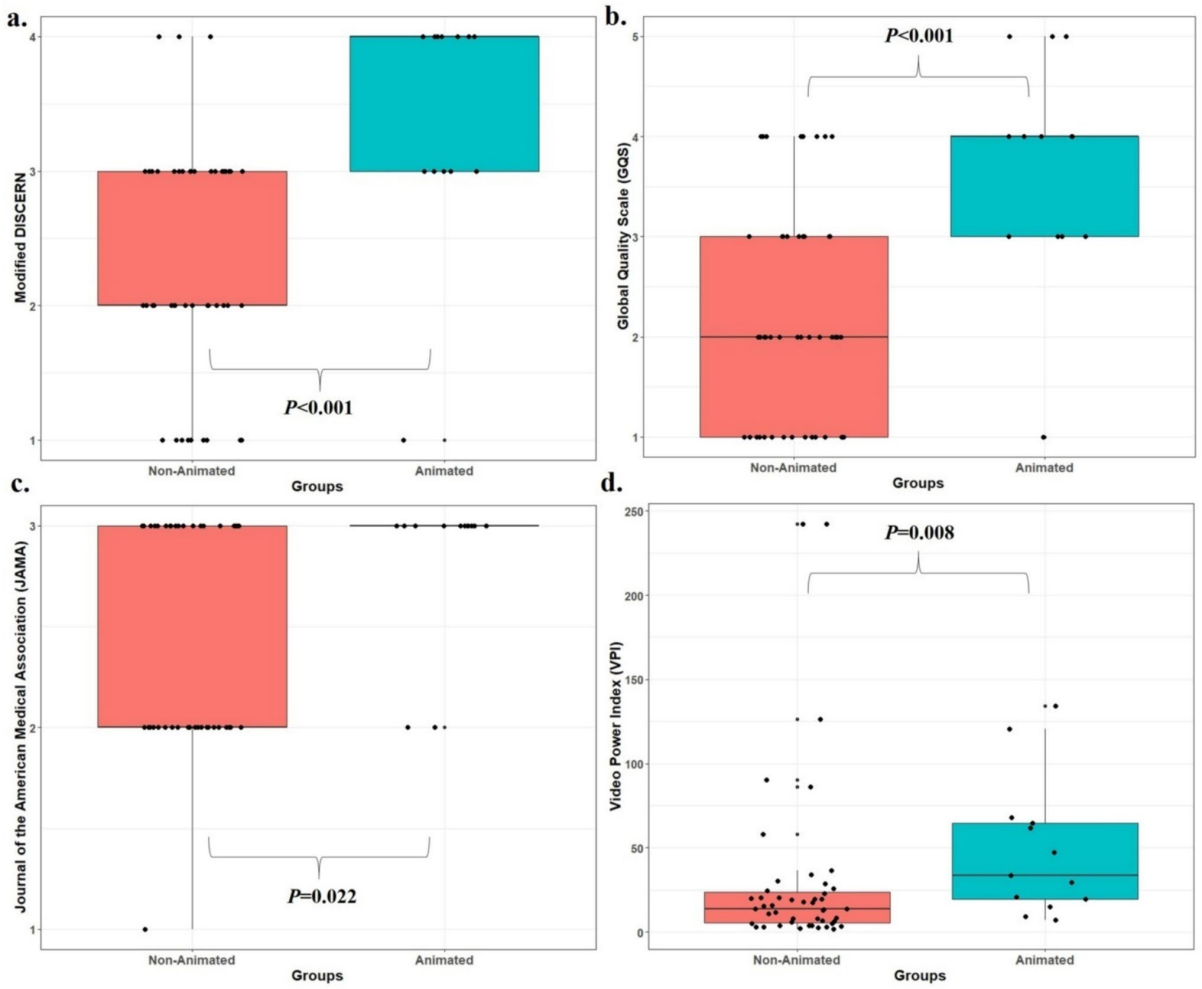
Boxplot with jitter showing the distribution of Global Quality Scale (GQS), Modified DISCERN, Journal of American Medical Association (JAMA) and Video Power Index (VPI) scores between animated and non-animated videos

As normality was not met between non-animated and animated video groups (mDISCERN: *P* < 0.001, *P* = 0.001; GQS: *P* < 0.001, *P* = 0.043; JAMA: *P* < 0.001, *P* < 0.001), the Mann–Whitney U test was used. Animated videos had significantly higher mDISCERN, GQS, JAMA, and VPI scores (*P* < 0.001, *P* < 0.001, *P* = 0.022, *P* = 0.008; Fig. 2), shorter durations (330 ± 187 s vs. 726 ± 799 s), more views (75,030 vs. 86,133), and higher VPI scores (48.5 ± 40.8 vs. 50.1 ± 177) compared to non-animated videos (*P* = 0.021, *P* = 0.008, *P* = 0.042, respectively).

**Fig. 3:**
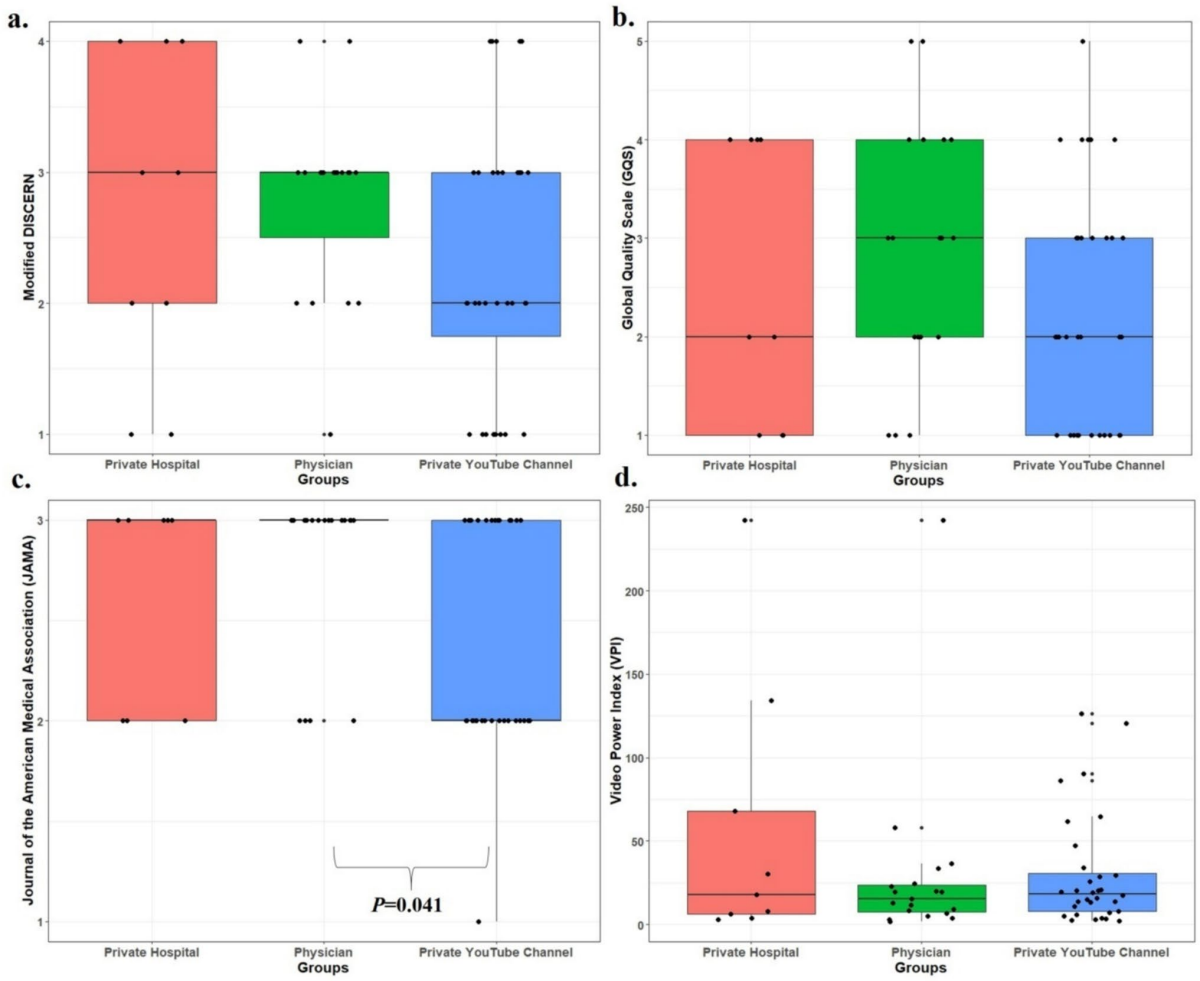
Boxplot with jitter showing the distribution of Global Quality Scale (GQS), Modified, Journal of the American Medical Association (JAMA) and Video Power Index (VPI) scores among video publishers

As normality was not met among the private hospitals, physicians, and private YouTube channel groups (mDISCERN: *P* = 0.122, 0.001, 0.002; GQS: *P* = 0.008, 0.144, 0.001; JAMA: *P* < 0.001 for all), comparisons were made using the Kruskal–Wallis test. Although private hospitals and physicians had higher mDISCERN and GQS scores than private YouTube channels, differences were not significant (*P* = 0.257, *P* = 0.248; Fig. 3). JAMA scores differed significantly among groups (*P* = 0.047); post-hoc analysis showed physicians' videos scored higher than private channels (*P* = 0.041). No other group differences were significant (*P* > 0.05). Viewing duration, views, likes, and VPI also showed no significant differences (*P* = 0.103, 0.792, 0.998, 0.834).

As normality was not met for mDISCERN, GQS, JAMA, video length, view count, likes, comments, VPI (all *P* < 0.001), and upload time (*P* = 0.001), Spearman correlation was used. A weak but significant negative correlation was found between upload time and mDISCERN, GQS, and JAMA scores (*P* = 0.007, 0.046, 0.018; Table 2). VPI scores showed a weak positive correlation with all three scales (*P* = 0.014, 0.048, 0.008). No correlation was observed between likes and mDISCERN or GQS (*P* > 0.05), but a weak positive correlation existed with JAMA (*P* = 0.020). Video length, views, and comments were not significantly associated with any scale scores (*P* > 0.05).

**Table 2:** Correlation analysis results identifying the relationships between video metrics and Modified DISCERN, GQS, and JAMA scores (n = 60)

		Modified DISCERN	GQS	JAMA
Video length (seconds)	r	.041	.028	.111
	*P*	.756	.833	.397
Time since upload (days)	r	− .344**	− .269*	− .304*
	*P*	**.007**	**.046**	**.018**
Number of views	r	.184	.138	.221
	*P*	.160	.294	.090
Number of likes	r	.230	.069	.299*
	*P*	.078	.602	**.020**
Comments	r	− .010	− .090	.119
	*P*	.943	.528	.402
VPI	r	.314*	.274*	.337**
	*P*	**.014**	**.048**	**.008**

*GQS* global quality scale, *JAMA* Journal of the American Medical Association, *VPI* Video Power Index

^*^Statistically significant at *P* < 0.05; ^**^Statistically significant at *P* < 0.01

A weak positive correlation was detected between video length and both the number of likes and the number of comments (*P* = 0.013, *P* = 0.005, respectively, Table 3), but no significant relationship was found between view count and VPI scores (*P* > 0.05, Table 3). A moderate negative correlation was identified between the time since a video’s upload and both the number of likes and the VPI scores (*P* < 0.001, *P* < 0.001, respectively, Table 3), but no significant relationship was found between the time since upload and the view count or comment count (*P* > 0.05, Table 3).

**Table 3:** Correlation analysis results showing the relationships between video characteristics and metrics (n = 60)

		Video length (seconds)	Time since upload (days)
Number of views	r	− .080	− .024
	*P*	.543	.856
Number of likes	r	.320*	− .463**
	*P*	**.013**	** < .001**
Comments	r	.382*	− .214
	*P*	**.005**	.128
VPI	r	− .016	− .595**
	*P*	.905	** < .001**

*VPI* video power index

^*^Statistically significant at *P* < 0.05; ^**^Statistically significant at *P* < 0.01

Although surgery-related videos had higher mean mDISCERN, GQS, and JAMA scores compared to informational videos, these differences were not statistically significant. This suggests that the quality of video content was not significantly influenced by whether the focus was on surgical treatment or other informational content related to Hirschsprung’s disease.

## Discussion

In our study, the content analysis revealed a notable lack of surgical videos related to Hirschsprung disease (11.7%). Despite the availability of various surgical procedures, the limited resources on surgical procedures highlight the need for pediatric surgeons to share surgical videos focused on these operations. The distribution of the seven surgical videos analyzed in our study by procedure type was as follows: 4 Transanal SOAVE pull-through, 1 Duhamel’s pull-through surgery, 1 Transanal full-thickness pull-through, and 1 Swenson Procedure. Additionally, the majority of the videos were shared by private YouTube channels (53.3%), underscoring the limited contributions from healthcare professionals. Çoşkun and Demir (2024), in their analysis of 45 Turkish circumcision videos, reported a surgical video rate of 13.3% (6 surgical and 39 informative videos) [15]. Consistent with the literature, there is a notable shortage of surgical videos, especially for healthcare professionals, on pediatric surgery topics on YouTube. Karabay et al. (2021), in their analysis of 100 videos on hypospadias surgery, reported that the educational content of hypospadias surgery videos on YouTube was unsatisfactory. They emphasized the need for videos to provide detailed information on surgical steps, such as glanuloplasty, skin closure, flap transposition, urethroplasty, and suture materials [22].

The average duration of Hirschsprung’s disease videos was 640.5 ± 729.8 s (approximately 11 min) with a median of 404 s (41–4,339 s, approximately 7 min). In the literature, the video durations found in other video analyses are as follows: for undescended testis videos, the median duration was 269 s (11–2,484 s) [23]; for newborn male circumcision, 5.1 ± 3 min [24]; for circumcision, 213.5 ± 206 s [15]; for bowel management, 22 ± 28.4 min [25]; and for vesicoureteral reflux, 704.8 s [26]. The average duration of Hirschsprung’s disease videos was lower than the durations of bowel management and vesicoureteral reflux videos but higher than those of undescended testis, newborn circumcision, and general circumcision videos reported in the literature.

The average view count of Hirschsprung’s disease videos was 83,728 (median 25,383), and the mean VPI was 49.8 (median 17.7). In the literature, the average view counts and VPI values for other video analyses are as follows: for undescended testis videos, the median view count was 589; for newborn male circumcision, the median view count was 39,247 and the VPI was 182.6 ± 328.3; for general circumcision, the average view count was 73,862 and the VPI was 39.9 ± 40.85; for bowel management, the average view count was 144,806; for vesicoureteral reflux, the average view count was 25,598 and the mean VPI was 0.2 ± 1.1; and for nocturnal enuresis videos in Japanese, the median VPI was 1.58 [15, 22–27]. The average view count and video popularity of Hirschsprung’s disease videos were higher than those of undescended testis, general circumcision, vesicoureteral reflux, and nocturnal enuresis videos, but lower than those of bowel management and newborn male circumcision videos.

For Hirschsprung’s disease videos, the average mDISCERN score was 2.53, the average GQS score was 2.52, and the average JAMA score was 2.55 (Medians: 3 (1–4), 2 (1–5), and 3 (1–3), respectively). In the literature, other video analyses report scale scores as follows: for undescended testis videos, the averages were mDISCERN 2.25 ± 1.2, GQS 2.82 ± 1.1, and JAMA 1.4 ± 0.99; for newborn male circumcision, the averages were mDISCERN 2.9 ± 0.7 and GQS 2.9 ± 1.1; for vesicoureteral reflux, the median scores for JAMA, mDISCERN, and GQS were 3 (1–4), 3 (1–5), and 3 (1–5), respectively [23, 24, 26]. Overall, the scale scores in our study were similar to those in the literature. Only 26.7% of the 60 videos evaluated by the GQS scale in our study were of high quality. Furthermore, only 3 videos (5%) achieved a full score of 5 on the GQS scale. None of the videos received a full score on the mDISCERN or JAMA scales. In their analysis of 93 nocturnal enuresis videos, Toprak and Tokat (2021) reported that 58% (*n* = 54) were of low quality, 16.2% (*n* = 15) were of moderate quality, and 25.8% (*n* = 24) were of high quality according to the GQS scale [28]. Toprak and Tokat (2021) concluded that the majority of YouTube content on nocturnal enuresis was of low quality. In general, video analyses on other pediatric surgery topics also report that the majority of videos are of low quality [15, 26–28]. Our study findings indicate that a significant portion of Hirschsprung’s disease videos have low quality and reliability scores. The low scale scores highlight the need for pediatric surgeons to produce and share high-quality videos that are both informative and surgical. These videos should be medically accurate, based on reliable sources, and presented in an easily understandable manner to ensure that viewers access accurate information. The importance of accessing reliable and high-quality information is especially critical on widely used platforms like YouTube, particularly for medical content.

It is known that YouTube users searching for information on a health condition tend to prioritize videos with higher view counts. In their study on YouTube videos about newborn male circumcision, Zaliznyak et al. (2022) found that lower-quality videos were more popular [24]. They reported a weak, significant negative correlation between overall video popularity and DISCERN (*r* = − 0.297, *p* = 0.031) and GQS quality ratings (*r* = − 0.274, *p* = 0.048) [24]. In contrast, Toprak and Tokat (2021) found no significant correlation between VPI values and GQS, JAMA, or DISCERN scores [28]. Similar to the findings of Toksoz and Duran (2021) [26], our study found a weak positive correlation between all scale scores and VPI. However, our findings indicated that the most-watched videos had particularly low GQS, mDISCERN, and JAMA scores. The most-viewed video, with over 2 million views, received a GQS score of 1 and mDISCERN and JAMA scores of 2. The second most-watched video had the same scores as the most-viewed one. This suggests that view counts are not directly related to video quality. Patients and their relatives often turn to videos with high view counts, but the reliability of these videos' content should be questioned.

Another significant finding of our study is that, while surgical videos had numerically higher scale scores than informational videos, this difference was not statistically significant. While our study did not find a statistically significant difference in quality between surgery-related and informational videos, this does not necessarily imply that non-surgical content is sufficiently covered. Given the complexity of Hirschsprung’s disease—which involves diagnostic challenges, long-term postoperative care, and potential complications—there remains a need for more comprehensive and high-quality educational materials beyond surgical techniques alone. The relatively low overall scores on mDISCERN, GQS, and JAMA scales further highlight the opportunity for improvement in both surgical and non-surgical video content.

Although the majority of videos on the surgical treatment of Hirschsprung disease were uploaded by physicians (71.4%), most informational videos (56.6%) were uploaded by non-professional YouTube channels. Nonetheless, the lack of a statistically significant difference in scale scores between surgical and informational videos highlights the need for improvement in the quality of surgical videos. We especially recommend that pediatric surgeons create and share higher-quality, comprehensive surgical videos. Such videos would play a critical role in providing accurate information on the complex procedures involved in Hirschsprung surgery, not only for patients and their families but also for residents and healthcare professionals.

In our study, the absence of a significant quality difference between surgical and informational videos is a noteworthy finding. It is generally expected that surgical videos, being prepared by professionals and containing technical content, would receive higher quality scores. However, this may be related to the fact that such videos are less accessible and comprehensible to lay audiences. Therefore, unless the content of surgical videos is simplified to be more easily understood by the general public, they may not stand out in terms of perceived quality compared to informational videos. This suggests that the relationship between the target audience and the presentation style of the content should be further explored in future studies.

According to our findings, videos that include animations received higher scores on the mDISCERN, GQS, and JAMA scales, indicating their value in education and information dissemination. The significantly higher popularity of videos with animations suggests that users prefer informational videos that incorporate animations. Additionally, the lack of a significant relationship between the number of likes and the GQS and mDISCERN scores implies that viewers may prioritize factors such as animation or view counts over quality. This finding could be attributed to several factors. Firstly, animations are known to enhance the clarity of complex medical concepts, making them easier to understand for a wider audience. By breaking down intricate information into visually engaging and digestible content, animations can improve the viewer's comprehension. Secondly, animations have the potential to increase engagement by capturing the viewer’s attention more effectively than static videos. This higher level of engagement may contribute to better retention and understanding of the material. Lastly, animations are often produced by reputable institutions with high standards of accuracy and reliability, which may further enhance their perceived trustworthiness. These factors combined likely contribute to the higher quality ratings observed for animated videos.

Although there was no significant difference in GQS and mDISCERN scores by publisher, the JAMA scores of videos uploaded by physicians were significantly higher than those uploaded by private YouTube channels. However, the low proportion of videos uploaded by physicians (31.7%) suggests that more video contributions from pediatric surgeons on Hirschsprung disease would positively enhance accurate and reliable information sources for patients, their families, and healthcare professionals. Consistent with our findings, Toksoz and Duran (2021) reported that videos on vesicoureteral reflux uploaded by hospitals and clinicians had significantly higher DISCERN, GQS, and JAMA scores compared to those uploaded by individual users and private YouTube channels [26].

Correlation analysis findings indicate a relationship between the content quality of videos and their publication date. Specifically, the weak yet significant negative correlation between mDISCERN, GQS, and JAMA scores and upload dates suggests that older videos tend to have lower quality according to current content standards. Therefore, we believe viewers should consider both content quality and publication date when selecting videos.

To facilitate a comprehensive comparison and provide context for our findings, we included previously studied conditions that are commonly encountered in pediatric surgery. These conditions represent topics for which YouTube video content quality analyses have already been conducted in the literature. Therefore, they were selected to allow for meaningful benchmarking against existing studies. This selection provides valuable insights into the consistency of video content quality across different disease groups. Additionally, the comparison among these conditions serves as an important basis for evaluating how both audiences and content creators perceive the quality of educational materials presented for more common diseases.

In light of these findings, it is imperative to consider practical implications for improving the quality of health-related content on digital platforms. Healthcare professionals, medical societies, and academic institutions should be encouraged to take a more active role in the production and dissemination of high-quality, evidence-based educational videos. Such content should ideally be developed in collaboration with interdisciplinary teams, including clinicians, educators, and digital communication experts, to ensure both medical accuracy and accessibility. Moreover, professional associations could establish partnerships with online platforms to endorse or highlight trustworthy videos, thereby guiding viewers toward more reliable information. These strategic efforts may contribute significantly to enhancing the overall quality and credibility of publicly available health information.

To our knowledge, this study is the first comprehensive assessment of the quality and content of YouTube videos on Hirschsprung disease. Our study provides valuable insights into the quality of these resources for both patient families and healthcare professionals. However, a limitation of our study is that our analyses are restricted to videos on the YouTube platform, as related content may also exist on other video platforms. The second limitation is that our study focused on general quality assessment rather than a detailed content analysis of specific subtopics (e.g., diagnostics, nutrition, or complications). Future studies may focus on more specific aspects of Hirschsprung’s disease, such as diagnostic procedures, nutritional management, postoperative care, or potential complications, to provide a more detailed evaluation of video content and educational value. The third limitation is that, While ICC values showed excellent inter-rater agreement (0.91–0.93), subjective biases in quality scoring (e.g., GQS’s reliance on perceived ‘usefulness’) persist. Future studies could incorporate patient feedback to validate and strengthen the reliability of these evaluation scales.

## Conclusion

This study revealed that YouTube videos related to Hirschsprung's disease are generally of low quality and that view counts do not align with content quality. It was observed that videos, mainly uploaded by private YouTube channels, provide limited information, especially on surgical procedures. Healthcare professionals, patients, and their families should focus on the reliability and scientific accuracy of content rather than view counts. Furthermore, it is recommended that pediatric surgeons, associations, or hospitals prepare comprehensive videos on Hirschsprung's disease that are both informative and surgery-oriented, incorporating educational elements like animations to support access to accurate information in society. In this way, patients and their families can make informed decisions and be better prepared for surgical processes.

## Data Availability

No datasets were generated or analysed during the current study.
